# A case report of gallbladder cancer and pancreas cystic neoplasm associated with pancreaticobiliary maljunction

**DOI:** 10.1016/j.ijscr.2021.106170

**Published:** 2021-07-07

**Authors:** Kazuhito Sato, Eisaku Ito, Yukiyoshi Masaki, Masako Ogawa

**Affiliations:** a The Department of Surgery, Ome Municipal General Hospital, 4-16-5 Higashi Ome, Ome City, Tokyo, Japan; b The Department of Pathology, Ome Municipal General Hospital, 4-16-5 Higashi Ome, Ome City, Tokyo, Japan; c The Department of Surgery, the Fraternity (Doai) Memorial Hospital, 2-1-11 Yokoami, Sumida-ku, Tokyo, Japan

**Keywords:** CT, computed tomography, FDG-PET, fluorine-18-fluorodeoxyglucose-positron emission tomography, IPMC, intraductal papillary mucinous carcinoma, IPMN, intraductal papillary mucinous neoplasm, MRCP, magnetic resonance cholangiopancreatography, MUC6, mucin type 6, PBM, pancreaticobiliary maljunction, Case report, Pancreaticobiliary maljunction, Gallbladder cancer, Intraductal papillary mucinous neoplasm

## Abstract

**Introduction and importance:**

Pancreaticobiliary maljunction (PBM) is a rare congenital anomaly that is frequently associated with carcinoma of the biliary tract. However, there is still no clear evidence that PBM is associated with pancreatic tumors. Here we describe a case of gallbladder cancer and intraductal papillary mucinous neoplasm (IPMN) that is associated with PBM.

**Case presentation:**

A 72-year-old man underwent a cholecystectomy with hepatectomy (S4a + S5) and regional lymph node dissection for gallbladder adenocarcinoma invading the front lobe branch of the hepatic artery. A pylorus-preserving pancreaticodudenectomy was also performed for pancreatic IPMN.

**Clinical discussion:**

Presence of mucin type 6 (MUC6) -positive pyloric gland metaplasia in both the dilated pancreatic duct and the gallbladder background mucosa suggests that pancreatic IPMN and gallbladder cancer may have a common phenotypic origin. Additionally, analysis of 41 reported cases of pancreatic cancer associated with PBM revealed that in all metachronous multiple cancer cases, biliary tract cancer preceded the pancreatic cancer with congenital biliary dilatation accompanied by PBM. The analysis also revealed an increased proportion of pancreatic cancer cases with PBM in patients who had not undergone a flow diversion procedure located in pancreatic head.

**Conclusion:**

We show an interesting relationship between pancreatic/gallbladder cancer and PBM. More comprehensive evaluations of the whole pancreaticobiliary system in follow-up of patients with PBM is required to understand the full extent of this relationship.

## Highlights

• Pyloric gland metaplasia seen both in the pancreatic duct and in gallbladder suggests that IPMN and gallbladder cancer may have a common origin.
• The analysis of pancreatic cancer with PBM cases revealed that in all metachronous cases, biliary tract cancer preceded the pancreatic cancer.
• This observation suggests that PBM patients should be monitored for synchronous or metachronous cancer of the whole pancreaticobiliary system.

## 1 Introduction

Pancreaticobiliary maljunction (PBM) is a congenital anomaly with an overall incidence of 0.03% in patients who have undergone endoscopic retrograde cholangiopancreatography [1] and 3.3% in patients who have undergone hepatobiliary tract surgery [2]. This anomaly occurs when the junction of the pancreatic duct and the common bile duct is located outside the sphincter of Oddi [3,4]; and is frequently associated with carcinoma of the biliary tract [3,5,6,8]. It has been proposed that biliary tract cancer develops when phospholipase A2 in refluxing pancreatic juice converts the phosphatidylcholine in bile into cytotoxic lysophosphatidylcholine. This can lead to damage of the epithelium with subsequent progression to carcinoma at the injured site [7]. Although PBM leads to the reciprocal regurgitation of pancreatic juice and bile, the association between pancreatic cancer and PBM is rare, possibly because the pancreatic duct pressure is higher than that of the bile duct [6,8,9].

Intraductal papillary mucinous neoplasms (IPMNs) were first described by Ohashi, et al. in 1982 [10], and Sessa, et al. introduced the term of IPMN in 1994 [11]. IPMN is characterized by the growth of epithelial tissue and mucin production in the main pancreatic duct or its branches [12]. It is agreed that IPMN is a precursor lesion of pancreatic carcinoma [11]; however, there is no evidence of a correlation between PBM and the development or malignant progression of IPMN. Here, we report a case of gallbladder cancer and IPMN associated with PBM. We used immunohistochemistry to show that PBM is involved in the development of the IPMN. We also performed a literature review of 41 cases of pancreatic neoplasm associated with PBM and show that in all metachronous cases, biliary tract cancer preceded the pancreatic cancer with congenital biliary dilatation accompanied by PBM. The case is reported according to SCARE criteria [13].

## 2 Presentation of case:

**Fig. 1:**
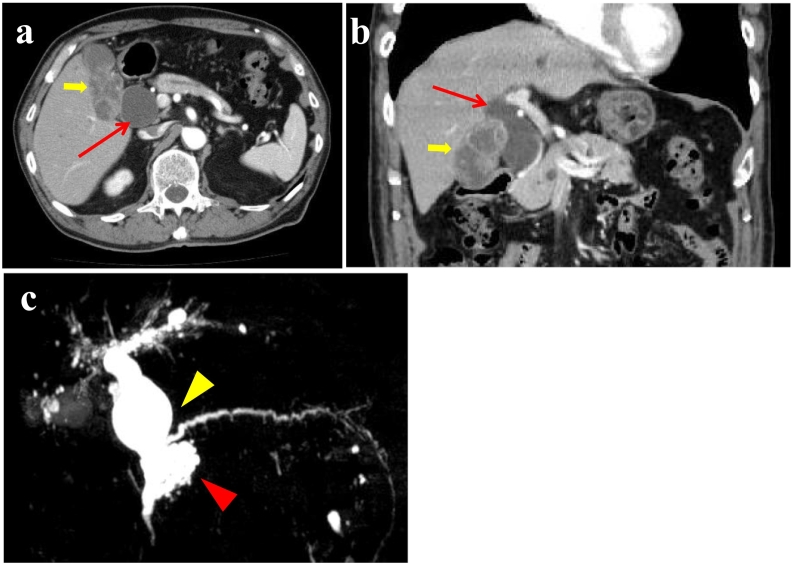
Preoperative CT and MRI. a-b A horizontal image (a) and a coronal image (b) of an enhanced CT scan showing enhanced wall thickness in the gallbladder (yellow arrow). The common bile duct was dilated up to 43 mm (red arrow). c A MRCP scan showed an anomalous junction of pancreaticobiliary tracts. Both the common bile duct and the intrahepatic bile duct are dilated (yellow arrowhead). The pancreatic uncinate process is occupied by a “bunch of grapes” lesion apart from main pancreatic duct suggesting the branch duct type IPMN (red arrowhead). (For interpretation of the references to colour in this figure legend, the reader is referred to the web version of this article.)

**Fig. 2:**
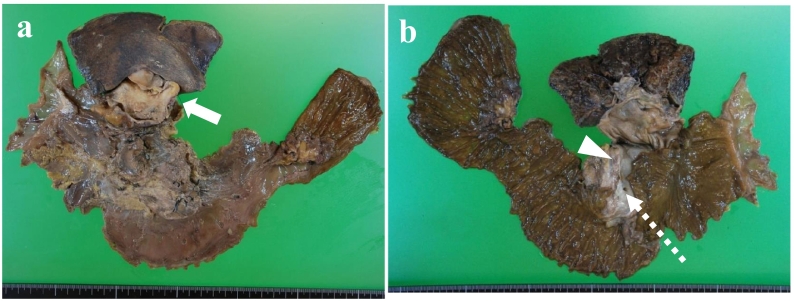
Macroscopic findings of the resected specimen. a Mucosal side showed the wall thickness of gallbladder (solid arrow). b Serosal side showed the cystic dilatation of the common bile duct (arrowhead). Pancreatic duct joined the common bile duct approximately 30 mm above the papilla of Vater (white dashed arrow).

A 72-year-old Japanese male patient was referred from a local clinic to our hospital suspected of gallbladder and pancreatic tumor. He had past medical history of hypertension treated with a calcium blocker. He had no other medical histories including psychiatric disorders. His family medical histories and genetic background do not report any relevant conditions. No particular physical findings were detected on admission. His laboratory test results are shown in Table 1. A computed tomography (CT) scan revealed a thickened gallbladder wall with enhancement, suggestive of gallbladder cancer invading the liver bed. The common bile duct showed Todani type Ia cystic dilatation (Fig. 1a, b). A fluorine-18-fluorodeoxyglucose-positron emission tomography (FDG-PET) scan revealed abnormal uptake of FDG in the gallbladder wall, supporting the gallbladder cancer diagnosis. No other abnormal FDG accumulation was detected. A magnetic resonance cholangiopancreatography (MRCP) scan showed that the pancreatic duct joined the common bile duct above the papilla of Vater, diagnosed as pancreaticobiliary maljunction type B (Fig. 1c). An aggregation of small cystic lesions in the pancreatic head and a slight dilatation of the pancreatic duct was detected which was compatible with the branch duct type IPMN (Fig. 2). The patient was diagnosed with a gallbladder adenocarcinoma and pancreatic IPMN and the case was discussed in the multidisciplinary conference including the tolerability to the procedure.

**Table 1:** Laboratory data.

Test item	Result	Normal range
Aspartate aminotransferase (AST)	17 IU/L	10–40
Alanine aminotransferase (ALT)	12 IU/L	5–45
Total bilirubin (T. bil)	0.7 mg/dL	0.3–1.2
Direct bilirubin (D. bil)	0.1 mg/dL	<0.4
Alkaline phosphatase (ALP)	224 IU/L	104–338
Gamma-glutamyl transferase (gamma-GTP)	23 IU/L	<79
Carcinoembryonic antigen (CEA)	3.4 ng/mL	<5.0
Carbohydrate antigen 19-9 (CA19-9)	75.4 U/mL	<37.0

### 2.1 Surgical findings

The patient consented to receiving the operation and underwent a cholecystectomy with hepatectomy (S4a + S5) and regional lymph node dissection for gallbladder cancer invading the front lobe branch of the hepatic artery. A pylorus-preserving pancreaticoduodenectomy was performed for pancreatic IPMN.

### 2.2 Pathological findings

**Fig. 3:**
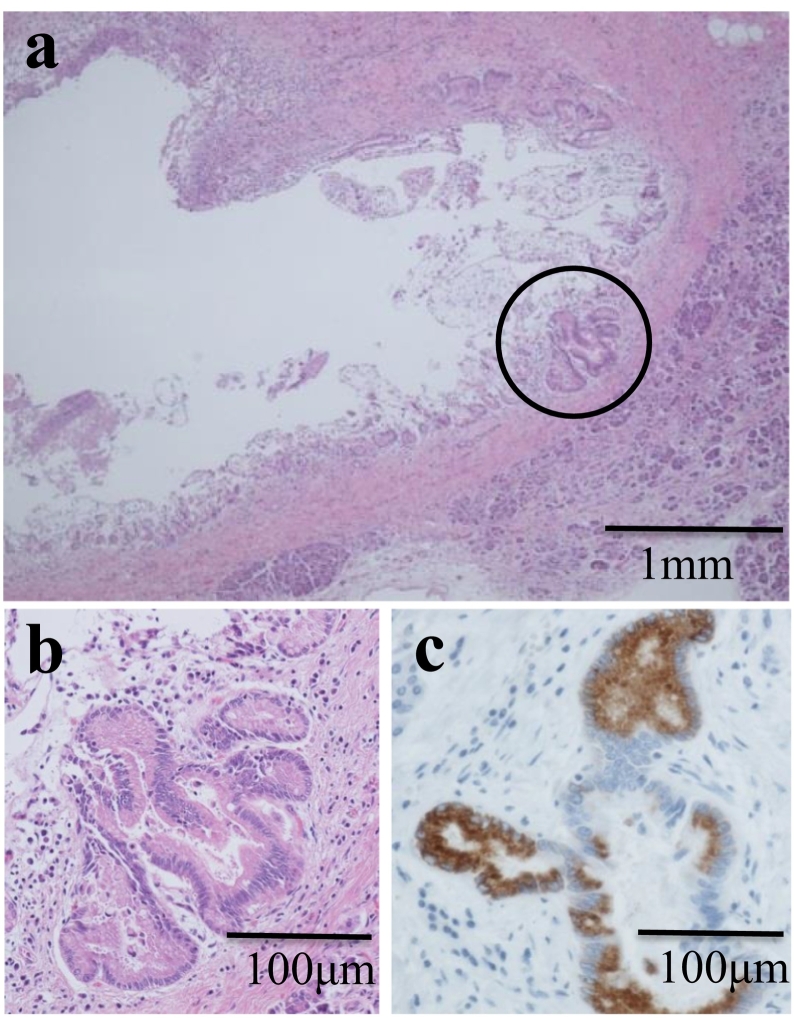
Histopathological examination of pancreas. a Hematoxylin and eosin (H&E) staining for the dilated pancreatic duct at the site of the pancreaticobiliary junction. The magnification of the micrograph is 40×. b Higher magnification (200×) for the circled area of panel a. Atypical changes were detected in the epithelium of the dilated pancreatic duct. c Immunohistochemistry with the anti-MUC6 antibody on the dilated pancreatic duct. The presence of MUC6-immunoreactive cells suggested pyloric gland metaplasia in the atypical pancreatic epithelium. The magnification of the micrograph is 200×.

**Fig. 4:**
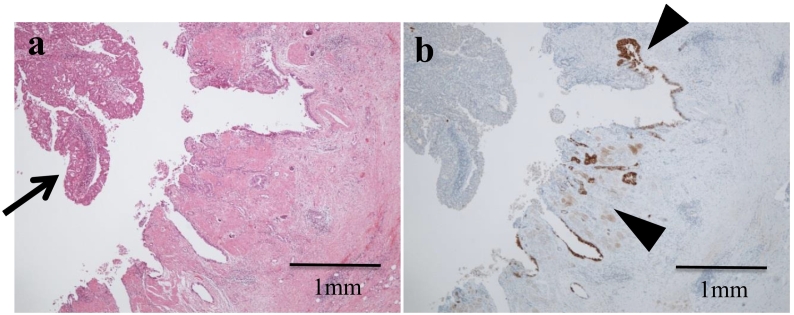
Histopathological examination of gallbladder. a Pathological diagnosis of gallbladder cancer was poorly differentiated tubular adenocarcinoma (arrow). The magnification of the micrograph is 40×. b Immunohistochemistry with anti-MUC6 antibody in the gallbladder. The gallbladder cancer lesion was negative for MUC6, whereas MUC6-positive pyloric gland metaplasia was detected in the non-cancerous mucosa of the gallbladder (arrowhead). The magnification of the micrograph is 40×.

Macroscopic findings of the resected gallbladder specimen revealed the tumor invading the liver bed. The histopathological diagnosis was a poorly differentiated tubular adenocarcinoma without lymph node metastasis, i.e., stage III according to the Union for International Cancer Control classification (Figs. 2a, 4a). The gallbladder cancer was evaluated as curative resection. With regard to the IPMN, precise pathological evaluation was impossible due to the autolysis of the surgical specimen. The main pancreatic duct at the pancreaticobiliary junction, adjacent to the IPMN, was dilated (Fig. 3a) and was associated with atypical changes (Fig. 3a, b).

Immunohistochemistry for mucin type 6 (MUC6) using the anti-MUC6 antibody was performed on the sections representing the pancreatic duct and the gallbladder cancer. The metaplastic lesion of the dilated main pancreatic duct was positive for MUC6, suggesting pyloric gland metaplasia (Fig. 3c). The gallbladder cancer lesion was negative for MUC6, whereas the non-cancerous background mucosa was positive, consistent with pyloric gland metaplasia (Fig. 4a, b).

## 3 Postoperative course and follow up

The patient was complicated with intraabdominal abscess which was treated with intravenous antibiotics administration. Furthermore, gastric bleeding caused by the compression of the drainage tube occurred 12 days after surgery, treated by parenteral hyperalimentation (Grade II according to Clavien-Dindo classification). The patient discharged the hospital postoperative day 69.

The patient was followed up with every 2 months after discharge. A CT scan taken 12 months after surgery revealed multiple metastasis in the hepatic hilum and perigastric lymph nodes, which was treated with gemcitabine+cicplatin chemotherapy. After 13 cycles, the regimen was changed to S1 due to nausea. He gave up receiving chemotherapy for the duration of nausea after 1 cycle of S1 and was followed up by a local clinic. He died 36 months after surgery.

## 4 Discussion

In this study we report a case of gallbladder cancer and IPMN that is associated with PBM. We show that MUC6-positive pyloric gland metaplasia is present in the dilated pancreatic duct as well as in the gallbladder background mucosa, suggesting that IPMN and gallbladder cancer may have a common origin.

Histopathological examinations have shown that PBM significantly increases incidences of hyperplastic changes in the non-cancerous epithelia of the gallbladder [14], [15] and of metaplasia and dysplasia in the biliary mucosa [15], [16]. Additionally, PBM is frequently associated with biliary tract cancer [3], [5], [6], [8], [7]. These results suggest that a sequence of hyperplastic changes through metaplasia and dysplasia plays an important role in the carcinogenesis of gallbladder with PBM. Although PBM induces the reciprocal regurgitation of bile and pancreatic juice, there are fewer reported cases of pancreatic cancer accompanied by PBM than biliary tract cancer [17], possibly because the pancreatic duct pressure is usually higher than that of the bile duct. However, after meals, bile duct pressure is raised by the contraction of the gallbladder, resulting in bile flowing into the pancreatic duct [8]. Pancreatic enzymes activated by the bile reflux possibly induce chronic inflammation and metaplastic epithelial change in the pancreatic duct, where pancreatic cancer may eventually develop [18].

We reviewed and summarized 41 reported cases of pancreatic cancer with PBM (Table 2). There were five cases with synchronous double cancers, two with synchronous triple cancers, six with metachronous double cancers, and one with metachronous triple cancers of the pancreas and the biliary tree. In all the metachronous cases, biliary tract cancer preceded the pancreatic cancer. Flow diversion procedure was performed for preceding gallbladder cancer in cases 14, 26, and 34. With the exception of these three cases and the cases with unknown history of diversion procedures, pancreatic cancer was located in the head or the entire pancreas in 25 of the 32 cases. This dataset indicated a trend of pancreatic cancer with PBM in patients who had not undergone a diversion procedure. In this subset of patients the pancreatic cancer was located in the pancreatic head, possibly due to the exposure to the regurgitated bile. However, data from the Japanese pancreatic cancer registry suggests that pancreatic head cancer had a higher instance than body/tail cancers by a ratio of 7:2 regardless of the etiology [57]. Therefore, whether bile regurgitation into the pancreatic duct affects the carcinogenesis of pancreatic cancer in PBM cases has yet to be confirmed. In terms of the pathology of the pancreatic tumors, at least five of the cases were reported to be intraductal papillary mucinous carcinoma (IPMC).

**Table 2:** Summary of the 41 reported cases of pancreatic cancer with PBM.

	Author	Ref	Year	Age/sex	PBM type	Todani class.	Locus of panc. ca.	Pathol. of panc. ca.	Complication of biliary tract ca.	Treatment	Survival time (month)
1	Dexter	[19]	###	22F	B	Ib	Head	Pap. ca.	Hepatic duct ca. (syn.)	Supportive care	24mo. death
2	Kelly, et al.	[20]	###	30M	Unk	I	Head	Adenoca.	Not described	Choledochoduodenostomy	6mo.
3	Binks, et al.	[21]	###	15M	Unk	I	Head	Unk	Not described	Choledochojejunostomy	3mo. death
4	Wood, et al.	[22]	###	34F	Unk	I	Head	Mucin-producing adenoca.	Not described	Inoperable	4mo. death
5	Deeg, et al.	[23]	###	70F	Unk	I	Body	Adenoca.	Not described	Inoperable, irradiation	6mo. death
6	Sanbonmatsu, et al.	[24]	###	15M	B	I	Whole	IPMC or MCC	None	Choledochoduodenostomy⇒choledochectomy and +hepaticojejunostomy→inoperable	3mo. death
7	Yoshitake, et al.	[25]	###	63M	A	ND	Head	Well-diff. tubular adenoca.	None	PD	Unk
8	Suda, et al.	[26]	###	66M	B	Unk	Head	Tubular adenoca.	Not described	PD	Unk
9	Kamisawa, et al.	[27]	###	83F	Unk	ND	Head	Well-diff. tubular adenoca.	Not described	Unknown	3mo. death
10	Ueda, et al.	[28]	###	58M	B	Ic	Whole pancreatic duct	Intraductal pap. adenoca.	GB, intrapancreatic bile duct (syn.)	Extended cholecystectomy + TP	30mo. alive
11	Aoki, et al.	[29]	###	74M	B	Ia	Whole	Mod.- diff. tubular adenoca.	None	PTCD	7mo. death
12	Kunimura, et al.	[30]	###	40F	C	Ic	Body	Intraductal pap. adenoca.	GB (met. 24mo.)	Extended cholecystectomy → DP R1	48mo. alive
13	Morohoshi, et al.	[31]	###	67F	Unk	Ic	Head	Intraductal pap. adenoca.	GB, bile duct (syn.)	PD + extended cholecystectomy	14mo. alive
14	Okada, et al.	[32]	###	63F	B	ND	Tail	cystadenocarcinoma	GB (met. 32mo.)	Extended right hepatectomy combined with bile duct resection →DP + T. mesocolon resection and left adrenalectomy	13mo. alive
15	Nakamura, et al.	[33]	###	70F	Unk	ND	Head	Por. diff. adenoca	GB (syn.)	Extended cholecystectomy+PD	Unk
16	Silas, et al.	[34]	###	75F	A	III	Head and uncinate	Adenoca.	None	Endoscopic drainage	4mo. death
17	Miura et al.	[35]	###	20F	A	Ia	Body	Mod.- diff. tubular adenoca.	Not described	Choledochojejunostomy⇒exploratory laparotomy, intraoperative irradiation	6mo. death
18	Kitajima, et al.	[36]	###	72F	Unk	Ic	Head	Intraductal pap. adenoca.	Not described	PD	Unk
19	Tazawa, et al.	[37]	###	48F	B	ND	Head	Well-diff. tubular adenoca.	Not described	PD	28mo. alive
20	Kuga, et al.	[38]	###	71F	A	Ic	Head	Well diff. adenoca.	None	PD	6mo. died
21	Kuga, et al.	[38]	###	56M	B	ND	Whole	Unk	None	Inoperable	4mo. died
22	Ozawa, et al.	[39]	###	45F	A	I	Head	Intramucosal pap. adenoca.	None	PpPD	≥ 120mo. alive
23	Ozawa, et al.	[39]	###	51M	B	Ia	Head	Mod.- diff. tubular adenoca.	None	PpPD	11mo. death
24	Hunerbein et al.	[40]	###	67F	B	Ic	Head-body	Adenoca.	None	Inoperable	Unk
25	Obana, et al.	[41]	###	53M	B	ND	Tail	Por. diff. adenoca.	None	Inoperable	6mo. death
26	Mayumi, et al.	[42]	###	41F	A	Ic	Head and uncinate (multiple)	Mod.- diff. tubular adenoca.	GB (met. 12mo.)	S4S + S5 resection combined with bile duct resection + D2 LN dissection→PD	5mo. death
27	Arakawa, et al.	[43]	###	68M	Unk	ND	Whole pancreatic duct	IPMC	None	TP	≥108mo. alive
28	Eriguchi, et al.	[44]	###	42F	Unk	I	Head	Intraductal pap. adenoca.	None	Choledochal cyst excision with Roux-en-Y hepaticojejunostomy ⇒ PpPD	60mo. alive
29	Kurokawa, et al.	[45]	###	50F	Unk	Ia	Head	Well-diff. pap. adenoca.	None	Choledochal cyst resection and hepaticojejunostomy⇒PD ⇒ TP (margin+)	5mo. death
30	Kobayashi, et al.	[46]	###	71F	B	ND	Body-tail	scc, scirrhous	GB (syn.)	DP cholecystectomy partial duodenectomy and jejunal resection (palliative)	6mo. death
31	Mizutani, et al.	[47]	###	71F	A	III	Head	Mod.- diff. tubular adenoca.	None	PD	Unk
32	Arakura, et al.	[48]	###	74F	C	Ic	Common channel	IPMC	None	PpPD	≥18mo. alive
33	Takeda, et al.	[49]	###	53F	A	Ia	Head	Well-diff. pap. adenoca.	None	Cholecystectomy, choledochectomy, and hepaticojejunostomy→TP	5mo. death
34	Takeda, et al.	[49]	###	50F	B	Unk	Body-tail	Mod.- diff. tubular adenoca.	GB (met. 122mo.)	Cholecystectomy, choledochectomy, and Roux-en-Ycholedochojejunostomy → distal pancreatectomy, splenectomy, left adrenalectomy, and partial gastrectomy	≥44mo. alive
35	Lahmar, et al.	[50]	###	68F	B	I	Head	Adenosquamous ca.	GB, CBD (met. 45mo.)	Cholecystectomy→bisegmentectomy→PD	≥12mo. alive
36	Honda, et al.	[51]	###	67M	A	I	Head-common channel	IPMC, oncocytic type	None	PpPD	≥19mo. alive
37	Koizumi, et al.	[52]	###	76F	B	ND	Head	Adenoca.	GB (met. 7mo.)	Cholecystectomy, choledochectomy, and Roux-en-Y choledochojejunostomy → chemo.	Unk
38	Rungsakulkij, et al.	[53]	###	46F	B	I	Head	Well-diff ductal type adenoca.	GB (syn.)	HPD	≥10mo. alive
39	Kinowaki, et al.	[54]	###	50M	B	I	Head	IPMC	None	PD	Unk
40	Mori, et al.	[55]	###	72F	B	I	Head	Mod.- diff. ductal adenoca. + IPMA	GB (syn.)	SSPPD+extended cholecystectomy	8mo. alive
41	Okubo, et al.	[56]	###	54F	B	Unk	Body-tail	IPMC, pancreatobiliary type	GB (met. 36mo.)	TP	≥6mo. alive

Abbreviations used in the table above:

Adenoca., adenocarcinoma; ca., cancer; CBD; common bile duct, chemo, chemotherapy; class., classification, diff., differentiated; DP, distal pancreatectomy; F, female; GB, gallbladder; HPD, hepatopancreaticoduodenectomy; M, male; MCC, mucinous cystic carcinoma; met., metachronous; mo., months; mod., moderately; ND, Non dilated; panc., pancreatic; pap., papillary; por., poorly; PD, pancreaticoduodenectomy; PpPD, pylorus-preserving pancreaticoduodenectomy; PTCD, percutaneous transhepatic cholangial drainage; syn., synchronous; Ref, reference; SSPPD, subtotal stomach-preserving pancreaticoduodenectomy; scc, squamous cell carcinoma; TP, total pancreatectomy; Unk, unknown

Although IPMN is a known precursor lesion of pancreatic carcinoma [11,12], the pathogenesis of IPMN has not been elucidated [[10], [11], [12]]. There is no evidence indicating a correlation between PBM and the development or malignant progression of IPMN. In terms of the etiology, the gallbladder cancer in this case was presumably caused by PBM. Immunohistochemistry of the IPMN lesion could not be evaluated due to the autolysis of the surgical specimen. Therefore, to explore whether PBM was involved in the development of IPMN in this patient, we performed immunohistochemistry of the dilated pancreatic duct. At the site of the pancreaticobiliary junction, the dilated pancreatic duct was associated with cellular atypia and MUC6-positive pyloric gland metaplasia, possibly due to exposure to the regurgitated bile. Pyloric gland metaplasia was also detected in the background mucosa of the gallbladder. Since pyloric gland metaplasia is a precancerous lesion for gallbladder cancer, these data suggest that IPMN and gallbladder cancer may have a common phenotypic origin. However, the association of pancreatic neoplasms, including IPMN, with PBM still remains unclear due to insufficient data. Future, more comprehensive evaluations of the whole pancreaticobiliary system in follow-up of patients with PBM will improve our understanding of the relationship between pancreatic neoplasms and PBM.

## 5 Conclusion

The presence of MUC6-positive pyloric gland metaplasia in the dilated pancreatic duct and gallbladder background mucosa in this patient suggests that IPMN and gallbladder cancer may have a common origin.

The analysis of 41 reported cases of pancreatic cancer with PBM showed that biliary tract cancer preceded the pancreatic cancer in all the metachronous multiple cancer cases. This observation suggests that patients with PBM should be monitored for synchronous and metachronous cancer of the whole pancreaticobiliary system, such that the appropriate surgical procedure and postoperative follow-up can be selected.

## Ethical approval

The publication of this case was approved by the Ethics Committee of Ome Municipal General Hospital.

## Sources of funding

This research did not receive any specific grant from funding agencies in the public, commercial, or not-for-profit sectors.

## Author contribution

KS is the first author of this manuscript. MO is the corresponding author. KS, who had 6 years of experience as a surgeon at that time, did the procedure as an operator, supervised by YM and another chief surgeon. MO assisted the procedure. KS and MO collected the clinical data. EI and MO collected the pathological data. YM revised the manuscript. All authors read and approved the final manuscript.

## Guarantor

Masako Ogawa, M.D., Ph.D.

## Research registration number

Not applicable.

## Consent for publication

The patient consented to the publication of these features of his case, and his identity has been protected.

## Provenance and peer review

Not commissioned, externally peer-reviewed.

## Declaration of competing interest

The authors declare that they have no competing interests.
